# Association Between Pharyngeal Pooling and Aspiration Using Fiberoptic Endoscopic Evaluation of Swallowing in Head and Neck Cancer Patients with Dysphagia

**DOI:** 10.1007/s00455-019-09992-x

**Published:** 2019-03-13

**Authors:** Sorina R. Simon, Michelle Florie, Walmari Pilz, Bjorn Winkens, Naomi Winter, Bernd Kremer, Laura W. J. Baijens

**Affiliations:** 1 Department of Otorhinolaryngology, Head and Neck Surgery, Maastricht University Medical Center, P.O. Box 5800, 6202 AZ Maastricht, The Netherlands; 2 School for Oncology and Developmental Biology – GROW, Maastricht University Medical Center, Maastricht, The Netherlands; 3 Department of Methodology and Statistics, Maastricht University, Maastricht, The Netherlands; 4 Care and Public Health Research Institute – CAPHRI, Maastricht University Medical Center, Maastricht, The Netherlands

**Keywords:** Deglutition, Deglutition disorders, Pharyngeal pooling, Aspiration, Head and neck cancer, Fiberoptic endoscopic evaluation of swallowing

## Abstract

Postswallow pharyngeal pooling may be a risk factor for tracheal aspiration. However, limited literature shows the potential association between pharyngeal pooling and aspiration in head and neck cancer (HNC) patients. This study investigates the relationship between postswallow pharyngeal pooling and aspiration in HNC patients with oropharyngeal dysphagia. Furthermore, the effects of tumor stage, tumor location, and cancer treatment on aspiration were examined. Ninety dysphagic HNC patients underwent a standardized fiberoptic endoscopic evaluation of swallowing (FEES) using thin and thick liquid boluses. For each swallow, three visuoperceptual ordinal variables were scored: postswallow vallecular pooling, postswallow pyriform sinus pooling, and aspiration. Logistic regression analyses with correction for the location of pooling, tumor stage, tumor location, and cancer treatment were performed to explore the association between pooling and aspiration. No significant association was found between postswallow vallecular pooling and aspiration for thin liquid. However, severe versus mild-to-moderate postswallow vallecular pooling of thick liquid was significantly associated to aspiration. Similar results were seen after correction for the presence of pyriform sinus pooling, tumor stage, tumor location, or type of cancer treatment. This study showed a significant association between severe postswallow pyriform sinus pooling of thick liquid and aspiration, independent of the presence of vallecular pooling, tumor stage, tumor location, or cancer treatment. Concluding, location (valleculae versus pyriform sinuses), liquid bolus consistency (thin versus thick liquid), and amount of postswallow pharyngeal pooling (no pooling, mild/moderate pooling, severe pooling) have an influence on the probability of aspiration in dysphagic HNC patients, and they should be carefully considered during FEES, even in the absence of aspiration during the examination.

**Electronic supplementary material:**

The online version of this article (10.1007/s00455-019-09992-x) contains supplementary material, which is available to authorized users.

## Introduction

Oropharyngeal dysphagia (OD) is common among patients with head and neck cancer (HNC) with a prevalence of 60–75% [1]. In this population, OD can be a result of the disease itself or its treatment. Treatment-induced alterations that contribute to OD include xerostomia, lymphedema, fibrosis, and damage to neuromuscular structures. Furthermore, chemoradiotherapy and the presence of hypopharyngeal carcinoma are associated with an increased risk for late OD [2]. Common symptoms of swallowing impairment in HNC patients while eating include complaints of food ‘sticking’ in the throat, nasal regurgitation, coughing, or choking [3]. OD following oncologic treatment can lead to dietary restrictions, dehydration, malnutrition, aspiration pneumonia, and death [4–6]. Therefore, an evaluation of swallowing complaints in patients with OD of oncological origin is highly recommended. To evaluate the pharyngeal phase of swallowing, fiberoptic endoscopic evaluation of swallowing (FEES) is deemed a reliable, safe, and well-tolerated tool. It provides a direct, two-dimensional view of the pharyngeal surface anatomy with a clear visualization of the bolus path [7]. Using FEES, the safety (aspiration) and efficacy (pooling) of swallowing can be evaluated [8–10]. Several studies used FEES to evaluate the swallowing function, but few used this method to assess OD in the HNC population [11, 12]. The visualization of aspiration during swallowing assessment in HNC patients has received much attention due to its potentially severe consequences, including aspiration pneumonia, sepsis, and death. Therefore, aspiration of food or liquids in patients with HNC is a major concern, with a reported incidence rate ranging from 36 to 94% [13]. Factors associated with aspiration are advanced age and tumor stage, previous history of head and neck surgery, and/or chemotherapy [13]. As postswallow pharyngeal pooling is assumed to pose a risk for tracheal aspiration on the subsequent swallow, the detection of pooling during swallowing assessment is also becoming increasingly important [14]. Postswallow pharyngeal pooling is defined as any portion of the bolus remaining in the valleculae and/or pyriform sinuses after the swallow, and it is considered to be a sign of impairment of deglutition [15, 16]. Reduced base-of-tongue retraction with loss of contact to the posterior pharyngeal wall and incomplete cricopharyngeal relaxation can result in pharyngeal pooling. This in turn may lead to postswallow aspiration, as observed during a videofluoroscopic swallow study (VFSS) in HNC patients [15]. Yet there is only limited literature regarding the possible association between postswallow pharyngeal pooling and aspiration in patients with HNC. Previous studies demonstrated that postswallow pharyngeal pooling is associated with aspiration during FEES examination in patients with nasopharyngeal carcinoma treated with definitive radiotherapy [11, 12]. However, these studies only included patients with nasopharyngeal carcinoma, and therefore their findings cannot be generalized to the overall HNC population, although, Jung et al. found an association between the presence of vallecular pooling and aspiration in patients with HNC [13]. During their retrospective analysis, a different swallowing assessment tool (VFSS) without standardized bolus consistencies was used, and not all patients suffered from OD. To our knowledge, no studies have explored the potential association between postswallow pharyngeal pooling and aspiration in a more general HNC population using FEES. The purpose of this study was to determine the association between postswallow pharyngeal pooling and aspiration in HNC patients with OD, using a standardized FEES protocol. The influences of tumor stage, tumor location, and cancer treatment on the association were also investigated.

## Methods

### Participants

HNC patients with complaints of OD who underwent a FEES examination at the Maastricht University Medical Center outpatient clinic between 2009 and 2016 were enrolled in the study. The study sample comprised patients drawn from a routine outpatient clinic and they were invited to participate if the inclusion/exclusion criteria were met. Patients were included if HNC treatment (surgery, radiotherapy, chemoradiotherapy, or combinations—multimodality treatment) had been completed at least 6 months before the data collection and FEES examination, and if the disease was in a stable period (total remission, the absence of radiation mucositis, or severe odynophagia). None of the patients was receiving palliative care. The following exclusion criteria were applied: HNC and a concurrent neurologic disease; a Mini Mental State Examination score below 23; older than 85 years; having undergone a total laryngectomy; having a recurrent HNC or a second primary tumor, and osteoradionecrosis of the maxilla or mandible. Cancer staging according to the tumor, nodes, and metastasis (TNM) classification system was performed [17]. Informed consent was obtained from all patients in the outpatient clinic. The study protocol is classified as non-WMO dutiful according to the Dutch Medical Research Human Subjects Act (http://www.ccmo.nl/en/non-wmo-research).

### Swallowing Protocol

A standardized examination protocol used in the dysphagia outpatient clinic for regular health care was applied. The protocol included a clinical ear, nose, and throat examination comprising integrity of cranial nerves performed by a laryngologist, the Functional Oral Intake Scale (FOIS) [7], and a standardized FEES examination [18]. The FOIS is used as a standardized measurement in daily clinical practice in the outpatient clinic for OD (heterogeneous etiologies of OD are present, not only HNC). It is used as part of a structured interview (descriptive variable) to assess the level of oral (or nonoral) intake. FOIS scores range from one (‘nothing by mouth’) to seven (‘total oral diet with no restrictions’) [7]. The FOIS provides an overall picture of which patients use a modified texture diet or tube feeding, and which patients have a total oral diet without any restrictions. Thus, the FOIS was used as a descriptive diet variable in this study. During the FEES examination, two liquid bolus consistencies were administered. Patients were offered three trials of thin liquid followed by three trials of thick liquid. Each trial contained 10 cc of water (thin liquid) or applesauce (One 2 fruit^®^) (hereafter ‘thick liquid’) dyed with five percent methylene blue as described in previous papers [19–21]. The viscosities (measured at 25 degrees Celsius 50 s^−1^ of shear rate) of the thin and thick liquid boluses were 1 mPa s and 1200 mPa s, respectively. Furthermore, during the flow test thick liquid met the descriptive criteria for ‘moderately thick’ according to the International Dysphagia Diet Standardisation Initiative (IDDSI) [22]. For safety concerns (risk of severe aspiration), some liquid bolus consistencies were not administered to all patients. Therefore, only subjects who had at least one trial with thin liquid or thick liquid were included in the study. The tip of the flexible fiberoptic endoscope Pentax FNL-10RP3 (Pentax Canada, Mississauga, Ontario, Canada) was positioned just above the epiglottis in the ‘high position’ [18]. FEES images were obtained using a Xion SD camera, XionEndoSTROB E camera control unit (PAL 25 fps), and Matrix DS datastation with DIVAS software (Xion Medical, Berlin, Germany). The images were recorded on a DVD at 30 frames per second. Neither a nasal vasoconstrictor nor a topical anesthetic was administered to the nasal mucosa.

### FEES Outcome Variables

For each FEES swallow trial, three visuoperceptual ordinal variables were scored: postswallow vallecular pooling, postswallow pyriform sinus pooling, and aspiration [20, 21]. The term ‘pooling’ was defined as the amount of bolus remaining in the valleculae and/or pyriform sinuses after spontaneous clearing swallows. No distinction was made between right- and left-sided pooling. Postswallow pooling was evaluated after the last swallowing of the same bolus, i.e., after the last piecemeal deglutition swallow. Three-point ordinal scales (range 0–2) were used to capture pooling severity. The categorical rating scale comprises three levels of pooling severity: no pooling (0), mild-to-moderate pooling (1), and severe pooling (2). The description of each ordinal level of the severity of pooling is based on the perceptual judgment of the amount of bolus in relation to the size of the valleculae and/or pyriform sinuses. Postswallow vallecular pooling was scored as no pooling (‘0’), mild-to-moderate pooling (‘1’: filling of less than 50% of the valleculae), or severe pooling (‘2’: filling of more than 50% of the valleculae up to complete filling). Postswallow pyriform sinus pooling was scored as no pooling (‘0’), mild-to-moderate pooling (‘1’: filling of less than 50% of the pyriform sinuses), or severe pooling (‘2’: filling of more than 50% of the pyriform sinuses up to complete filling). Severe pooling in the valleculae means pooling up to the free edge of the epiglottis. For pyriform sinus pooling, severe pooling was up to the level of the arytenoids. Aspiration was defined as bolus passing below the level of the vocal folds entering the trachea. Bolus on the true vocal folds secondarily leaking in the trachea was also classified as aspiration. Therefore, bolus in and below the anterior commissure was scored as aspiration. These FEES outcome variables were described in previous studies and are presented in the supplementary material (Table S1) [20, 21]. Prior to data collection, two experts received consensus training on the interpretation of the ordinal FEES variables. This is a standardized procedure for each new study containing visuoperceptual assessment tools for OD. The protocol of this training has been described in previous studies [19, 21]. Both observers were blinded to the identity and medical history of the patient and to each other’s ratings (independent rating). The swallow trials were scored in randomized order at varying speeds (slow motion, normal, and up to frame-by-frame). To obtain intraobserver agreement, each observer performed repeated measurements of all visuoperceptual FEES variables. Moreover, observers were advised to limit the duration of the measurement sessions (maximum of two hours per session) to avoid fatigue-related bias.

### Statistical Analysis

Descriptive statistics were reported in terms of means with standard deviations (SDs) for numerical variables and number (percentage) for categorical variables. The score indicating more severe impairment of each FEES variable, dependent on liquid bolus consistency (thin or thick liquid), was used for statistical analysis. Where appropriate, a Chi-square test or Fisher’s exact test was used to analyze whether the proportions of events (aspiration) were different for various amounts of pooling. Logistic regression analysis was performed to further explore the association between pooling and aspiration. Correction for pooling in the other location (pyriform sinuses versus valleculae) was performed to determine whether pooling location was associated with aspiration, independent of pooling in the other location. The analysis was also adjusted for the influence of tumor stage (T3–4 vs. T1–2), tumor location (pharynx tumor versus non-pharynx tumor), and cancer treatment (only radiotherapy, only surgery, or multimodality treatment). Multimodality treatment refers to a combination of (primary or salvage) surgery, (neo)adjuvant radiotherapy, and/or chemotherapy [23]. Due to the limited number of events (aspiration) and nonevents (no aspiration), it was not possible to adjust for all three factors simultaneously, and thus, these factors were included in the analysis separately. Furthermore, the effect of tumor stage, tumor location, or cancer treatment on aspiration was determined. A two-sided *p* value ≤ 0.05 was considered statistically significant. For each visuoperceptual ordinal FEES variable, the inter- and intraobserver agreement levels were calculated using linear weighted Kappa coefficient [24]. All statistical analyses were performed using IBM SPSS Statistics for Windows, version 23.0 (IBM, Armonk, NY).

## Results

### Participants

Ninety patients (81.1% men) were included in the study. The mean age of the patients was 65.9 years (SD 10.8). All patients reported OD complaints. Patients’ characteristics including TNM classification, tumor location, oncologic treatment, and FOIS score are presented in Table 1. Pharynx (51.1%) and larynx (26.1%) were the most common tumor sites. Within the group of patients with pharyngeal cancer, oropharynx was the most common tumor location (60%). Squamous cell carcinoma of the head and neck was the most frequent type of cancer (76.7%). The majority of the patients (88.8%) underwent radiotherapy as single modality or part of multimodality treatment (Table 1). Fourteen patients (16.1%) underwent a tracheotomy. The mean score of the FOIS was 4.7 (SD 1.8). Seventy-six patients underwent the entire FEES examination protocol. For safety concerns (risk of severe aspiration), 14 patients did not receive the entire FEES protocol (3 × 10 cc thin, 3 × 10 cc thick liquid): 3 patients only received thin liquid, and 11 patients only received thick liquid.

**Table 1:** Frequency distributions of HNC patient characteristics (total number of patients = 90)

Characteristics	Number of patients (%)
Age distribution (*N* = 90)	
< 60 years	22 (24.4)
≥ 60 years	68 (75.6)
Length of time since completion of HNC treatment (*N* = 89)	
< 5 years after treatment	62 (69.7)
> 5 years after treatment	27 (30.3)
T classification (*N* = 75)	
T1	17 (22.7)
T2	25 (33.3)
T3	13 (17.3)
T4	20 (26.7)
N classification (*N* = 75)	
N0	37 (49.3)
N1	11 (14.7)
N2	25 (33.3)
N3	2 (2.7)
M classification (*N* = 75)	
M0	75 (100)
Tumor location (*N* = 88)	
Pharynx	45 (51.1)
Larynx	23 (26.1)
Oral cavity	11 (12.5)
Other location^a^	9 (10.2)
Treatment (*N* = 89)	
Definitive radiotherapy	37 (41.6)
Surgery	10 (11.2)
Surgery and adjuvant radio(chemo)therapy	26 (29.2)
Definitive radiochemotherapy	16 (18)
Tracheotomy (*N* = 87)	
Tracheotomy	14 (16.1)
No tracheotomy	73 (83.9)
FOIS (*N* = 88)	
Level 1	8 (9.1)
Level 2	9 (10.2)
Level 3	1 (1.1)
Level 4	5 (5.7)
Level 5	38 (43.1)
Level 6	14 (15.9)
Level 7	13 (14.8)

*HNC* head and neck cancer; *FOIS* functional oral intake scale

^a^Tumor sites including the nasal cavity or paranasal sinuses are reported as ‘Other location’

### Observer Agreement

The interobserver and intraobserver agreement levels were sufficient for all FEES variables (all Kappa coefficients ≥ 0.71, indicating substantial to almost perfect agreement) (Table S2 in supplementary material).

### Postswallow Vallecular Pooling

#### Thin Liquid Bolus Consistency

Postswallow vallecular pooling of thin liquid bolus consistency occurred in 39 (51.3%) patients: mild-to-moderate and severe vallecular pooling were observed in 24 (31.6%) and 15 (19.7%) patients, respectively (Table 2). Twenty-four (61.5%) of the patients showing postswallow vallecular pooling aspirated. ‘Mild-to-moderate vallecular pooling’ compared to ‘no pooling,’ was not significantly associated with aspiration (OR 1.94, 95% CI 0.69, 5.51, *p* = 0.212) (Table 3). However, ‘severe vallecular pooling’ compared to ‘no pooling’ was significantly associated with aspiration (OR 4.52, 95% CI 1.20, 16.97, *p* = 0.026). The effect of ‘severe vallecular pooling’ compared to ‘mild-to-moderate pooling’ on aspiration was not significant (OR 2.33, 95% CI 0.58, 9.43, *p* = 0.236). Correction for pooling in the other location was performed to determine whether a certain pooling location was associated with aspiration, independent of pooling in the other location. The effect of ‘severe vallecular pooling’ compared to ‘no pooling’ on aspiration appeared not significant (OR 2.58, 95% CI 0.56, 11.91, *p* = 0.226) after this correction (pooling in pyriform sinuses). Similar results were seen after correction for tumor stage, tumor location, or type of cancer treatment.

**Table 2:** Frequency distribution of the occurrence of postswallow pharyngeal pooling and aspiration (total number of patients = 90)

Pooling location and liquid bolus consistency	Presence of pooling^a^ *N* (%)	Aspiration in patients who presented pooling *N* (%)	Aspiration in patients who did not present pooling *N* (%)
Valleculae			
Thin liquid (*N* = 76)	39 (51.3)	24 (61.5)	14 (37.8)
Thick liquid (*N* = 79)	69 (87.3)	22 (31.9)	4 (40.0)
Pyriform sinuses			
Thin liquid (*N* = 77)	26 (33.8)	17 (65.4)	22 (43.1)
Thick liquid (*N* = 80)	38 (47.5)	20 (52.6)	8 (19.0)

*N* number of patients

^a^Presence of postswallow pooling is defined as the presence of mild-to-moderate pooling and/or severe pooling

**Table 3:** Association between aspiration and postswallow vallecular or pyriform sinus pooling

	Unadjusted^a^				Adjusted for location of pooling^b^			
	Thin liquid		Thick liquid		Thin liquid		Thick liquid	
	OR (95% CI)	*p*-value	OR (95% CI)	*p*-value	OR (95% CI)	*p*-value	OR (95% CI)	*p*-value
Postswallow vallecular pooling								
Mild-to-moderate pooling versus no pooling	1.94 (0.69, 5.51)	0.212	0.21 (0.04, 1.11)	0.066	1.39 (0.44, 4.35)	0.572	0.18 (0.03, 1.05)	0.056
Severe pooling versus no pooling	4.52 (1.20, 16.97)	0.026*	1.42 (0.34, 5.88)	0.628	2.58 (0.56, 11.91)	0.226	0.80 (0.17, 3.78)	0.781
Severe pooling versus mild-to-moderate pooling	2.33 (0.58, 9.43)	0.236	6.62 (1.94, 22.73)	0.003*	1.86 (0.41, 8.33)	0.420	4.51 (1.17, 17.54)	0.029*
Postswallow pyriform sinus pooling								
Mild-to-moderate pooling versus no pooling	1.13 (0.33, 3.84)	0.845	4.25 (0.87, 20.75)	0.074	0.92 (0.25, 3.42)	0.895	5.87 (1.00, 34.41)	0.05004
Severe pooling versus no pooling	7.25 (1.46, 36.10)	0.016*	4.86 (1.70, 13.91)	0.003*	4.78 (0.80, 28.35)	0.086	3.44 (1.03, 11.47)	0.044*
Severe pooling versus mild-to-moderate pooling	6.41 (0.99, 41.67)	0.0501	1.14 (0.24, 5.44)	0.867	5.21 (0.78, 34.48)	0.089	0.59 (0.10, 3.34)	0.548

*OR* odds ratio, *CI* confidence interval

^a^Logistic regression analysis, without adjustment for other variables

^b^Logistic regression analysis, with adjustment for pooling in the other location

*Significance at *p* ≤ 0.05

#### Thick Liquid Bolus Consistency

In 69 (87.3%) patients, postswallow vallecular pooling of thick liquid bolus consistency was observed and was scored as mild-to-moderate and severe pooling in 32 (40.5%) and 37 (46.8%) patients, respectively (Table 2). Aspiration occurred in 22 (31.9%) of the patients showing postswallow vallecular pooling. The analysis regarding amount of pooling in the valleculae showed that, when compared to ‘no vallecular pooling,’ both ‘mild-to-moderate pooling’ (OR 0.21, 95% CI 0.04, 1.11, *p* = 0.066) and ‘severe pooling’ (OR 1.42, 95% CI 0.34, 5.88, *p* = 0.628) were not significantly associated with aspiration (Table 3). However, the effect of ‘severe vallecular pooling’ compared to ‘mild-to-moderate pooling’ on aspiration was significant (OR 6.62, 95% CI 1.94, 22.73, *p* = 0.003). Similar results were seen after correction for pooling location, and after additional correction for tumor stage, tumor location, or type of cancer treatment.

### Postswallow Pyriform Sinus Pooling

#### Thin Liquid Bolus Consistency

Postswallow pyriform sinus pooling of thin liquid bolus consistency occurred in 26 (33.8%) patients: mild-to-moderate and severe pyriform sinus pooling were observed in 13 (16.9%) and 13 (16.9%) patients, respectively (Table 2). Seventeen (65.4%) of the patients showing postswallow pyriform sinus pooling aspirated. ‘Mild-to-moderate pyriform sinus pooling’ compared to ‘no pooling,’ was not significantly associated with aspiration (OR 1.13, 95% CI 0.33, 3.84, *p* = 0.845) (Table 3). However, ‘severe pyriform sinus pooling’ compared to ‘no pooling’ was significantly associated with aspiration (OR 7.25, 95% CI 1.46, 36.10, *p* = 0.016). Although a trend toward significance was seen, the effect of ‘severe pyriform sinus pooling’ compared to ‘mild-to-moderate pooling’ on aspiration was not significant (OR 6.41, 95% CI 0.99, 41.67, *p* = 0.0501). After correction for pooling in the other location (valleculae), the effect of ‘severe pyriform sinus pooling’ compared to both ‘no pooling’ (OR 4.78, 95% CI 0.80, 28.35, *p* = 0.086) and ‘mild-to-moderate pooling’ (OR 5.21, 95% CI 0.78, 34.48, *p* = 0.089) on aspiration was not found to be significant. Similar results were seen following additional correction for tumor stage or tumor location. An exception was found after correction for type of cancer treatment. The effect of ‘severe pyriform sinus pooling, compared to both ‘no pooling’ (OR 9.99, 95% CI 1.44, 64.47, *p* = 0.020) and ‘mild-to-moderate pooling’ (OR 11.24, 95% CI 1.37, 90.91, *p* = 0.024), on aspiration was found to be significant.

#### Thick Liquid Bolus Consistency

Postswallow pyriform sinus pooling of thick liquid bolus consistency was observed in 38 (47.5%) patients, which was scored as ‘mild-to-moderate’ and ‘severe pooling’ in 8 (10%) and 30 (37.5%) patients, respectively (Table 2). Aspiration occurred in 20 (52.6%) of the patients showing pyriform sinus pooling. ‘Mild-to-moderate pyriform sinus pooling’ compared to ‘no pooling’ was not significantly associated with aspiration (OR 4.25, 95% CI 0.87, 20.75, *p* = 0.074) (Table 3). However, the effect of ‘severe pyriform sinus pooling’ compared to ‘no pooling’ on aspiration was significant (OR 4.86, 95% CI 1.70, 13.91, *p* = 0.003). The effect of ‘severe pyriform sinus pooling’ compared to ‘mild-to-moderate pooling’ on aspiration was not significant (OR 1.14, 95% CI 0.24, 5.44, *p* = 0.867). Similar results were seen after correction for pooling in the other location, and after additional correction for tumor stage, tumor location, or type of cancer treatment.

### Effect of Tumor Stage, Tumor Location, or Cancer Treatment on Aspiration After Correction for Postswallow Pooling in Both Locations

After correction for postswallow pooling in valleculae and pyriform sinuses location, the effect of tumor stage, tumor location, or cancer treatment on aspiration was determined.

Both tumor stage (*p* = 0.764) and tumor location (*p* = 0.470) had no significant effect on aspiration in swallows of thin liquid bolus consistency. However, cancer treatment showed a significant effect on aspiration (overall *p* = 0.003). The proportion of patients with aspiration was significantly higher for the group of patients who only underwent definitive radiotherapy, compared to the group of patients who received multimodality treatment (OR 7.86, 95% CI 2.19, 28.23, *p* = 0.002). The group of patients who underwent surgery exclusively, did not have a significantly increased proportion of patients with aspiration, compared to the group of patients who received multimodality treatment (OR 0.79, 95% CI 0.12, 5.07, *p* = 0.800). Finally, tumor stage, tumor location, and cancer treatment had no significant effect on aspiration in swallows of thick liquid bolus consistency (all *p*-values ≥ 0.209).

## Discussion

In this study, we investigated the relationship between postswallow pharyngeal pooling and aspiration in dysphagic HNC patients using FEES. Furthermore, we analyzed the association between tumor stage, tumor location, and cancer treatment on the one hand, and aspiration on the other hand.

The results of our study showed that location, liquid bolus consistency, and amount of postswallow pharyngeal pooling have an influence on the probability of aspiration. ‘Severe vallecular pooling’ of thick liquid, compared to ‘mild-to-moderate pooling,’ showed a significant association with aspiration, independent of the presence of pooling in the pyriform sinuses. However, no significant association was found between ‘severe vallecular pooling’ of thick liquid versus absence of pooling and aspiration. This outcome is not what would be expected in clinical practice, and no clear explanation was found for it. It would be interesting to replicate this study with a larger sample size to verify if the association might change. Whereas, severe vallecular pooling of thin liquid, compared to the absence of pooling, was only significantly associated with aspiration if there was also pooling in the pyriform sinuses. This finding may be explained by the greater distance between the valleculae and laryngeal vestibule, compared to the anatomic position of the pyriform sinuses relative to the laryngeal vestibule [25]. As the bolus may spill from the lateral parts of the valleculae, the overflow of the bolus from the valleculae directly into the laryngeal vestibule seems less likely to happen. The epiglottis might play an additional role as a barrier between the valleculae and laryngeal vestibule, even though oncologic treatment or tumor location (oropharynx, supraglottis) in HNC patients may impair the epiglottic function due to fibrosis, malformation, and tissue destruction [26]. Consequently, in the case of severe vallecular pooling, this epiglottis barrier function may fail. Also the phenomena of pooling in both locations (pyriform sinuses and valleculae) probably indicate a more severe swallowing impairment with an increased risk of aspiration [27]. Previous studies by other authors demonstrated a significant association between vallecular pooling and aspiration [13, 16, 27]; however, most studies included mainly non-oncological patients [16, 27]. Considerable heterogeneity exists across studies making comparisons difficult, especially regarding the methodology and study population, as the majority of patients showed neurogenic OD [16, 27]. Only one study included patients with HNC [13]. Moreover, in previous studies the swallowing function was evaluated with a different assessment tool (VFSS), which may influence the results, considering that FEES may yield more severe scores for both pooling and penetration/aspiration, when compared to VFSS [28, 29]. Furthermore, nonstandardized bolus consistencies and/or varying volumes were administered across these studies [13, 16] and diverse rating scales for pharyngeal pooling were applied [16, 30].

In the current study, we found a significant association between severe pyriform sinus pooling versus the absence of pooling and aspiration of thin liquid, without correction for vallecular pooling in the statistical model. This finding may be related to the thin and watery nature of the boluses which can very quickly flow, but may not apply for thicker or solid boluses that stick and clump. However, after correction for vallecular pooling in the regression model no significant association was found between severe pyriform sinus pooling versus the absence of pooling and aspiration of thin liquid. In the current study, the presence of severe versus no pyriform sinus pooling of thick liquid was significantly associated with aspiration, independent of the presence of vallecular pooling. This association may be explained by the proximity of the pyriform sinuses to the laryngeal vestibule, facilitating the overflow of the bolus from the pyriform sinuses into the laryngeal vestibule [25]. Decreased opening of the UES, which has been previously observed in patients with HNC treated with (chemo)radiotherapy, impairs the passage of food or liquids from the (hypo)pharynx into the esophagus and may contribute to the occurrence of bolus overflow from the pyriform sinuses into the laryngeal vestibule [31]. More than 80% of the patients in our study received radiotherapy (single or multimodality treatment). Ku et al. also found a significant association between postswallow pyriform sinus pooling and aspiration assessed during FEES in dysphagic patients with nasopharyngeal carcinoma [11]. Other studies using VFSS mainly in patients with neurogenic OD did not show any association between pyriform sinus pooling and aspiration [16, 27]. However, some studies reported that the risk of aspiration increased with the increasing amounts of pharyngeal pooling [27, 32]. Likewise, our results showed that an increasing amount of postswallow pooling in the pyriform sinuses significantly increased the incidence of aspiration in HNC patients.

It was hypothesized that the type of treatment may contribute to the occurrence of postswallow pharyngeal pooling and/or aspiration as oncologic treatment can cause impaired pharyngeal contraction and laryngopharyngeal motorsensory deficits due to fibrosis and postradiation neuropathy [6]. Other factors, including an advanced tumor stage, were thought to strengthen the association between postswallow pharyngeal pooling and aspiration. However, in the present study, the association between postswallow pharyngeal pooling and aspiration did not change after correcting for tumor stage, tumor location, or type of cancer treatment, with the exception of a significant association between ‘severe pyriform sinus pooling’ of thin liquid and aspiration after correction for type of cancer treatment. The fact that an association between tumor stage and pooling or aspiration was not found might be a result of the small sample size: perhaps an association would have been found if more patients were included in the study. The distribution of tumor stage among the included patients (i.e., 42 patients with T1 or T2 stage versus 33 patients with T3 or T4 stage) is considered fairly equal. A more elaborate statistical analysis, including detailed group stratification for all (separate) tumor stages, might have led to different results. However, the present sample size was too small to allow statistical pooling.

Additionally, our results indicated that the occurrence of aspiration of thin liquid is influenced by cancer treatment, whereby patients who underwent definitive radiotherapy as a single modality treatment demonstrate significantly higher aspiration rates, compared to patients who received multimodality treatment, which comprises a combination of (primary or salvage) surgery, (neo)adjuvant radiotherapy and/or chemotherapy. According to the report of Pearson et al., HNC patients treated with radiotherapy demonstrated poor swallowing outcomes, including increased aspiration and pharyngeal pooling [33]. However, discordant results on aspiration have been reported in HNC patients too. Several studies found that aspiration was significantly associated with either tumor stage [4, 13, 34], tumor location [34, 35], or cancer treatment [13]. In contrast, other studies could not demonstrate a significant relation between either tumor stage [35], tumor location [4, 13], or cancer treatment [4] on the one hand, and aspiration on the other hand. It has also been suggested that the presence of aspiration in HNC patients is affected by the type of cancer treatment (surgery, chemotherapy, or radiotherapy), rather than by tumor location [13].

Our study investigated the potential association between postswallow pharyngeal pooling and aspiration using FEES in dysphagic HNC patients. However, it is unknown whether the association between postswallow pyriform sinus pooling and aspiration represents a high co-occurrence rate, or a causal association. Patients who showed significant pooling during FEES may not present aspiration during the examination, but they may aspirate after the examination or at any time, when they are eating at home in their daily habitat [19]. In fact, FEES with its standardized protocols is only a short observation of a complex swallowing process, and therefore it is not always a realistic representation of daily swallowing. In a nonclinical setting, multiple factors may affect the swallowing mechanism such as fatigue, increased complexity of feeding in terms of various bolus consistencies, and diverse eating behaviors and postures. Previous research in patients with OD showed that the risk of aspiration can be underestimated when a limited number of swallow trials is performed during FEES [19]. Based on our results, we assumed that patients presenting severe pyriform sinus pooling of thick liquid are more likely to present aspiration, compared to patients who do not present pooling in the pyriform sinuses. Thus, pyriform sinus pooling in HNC patients is not only a marker of impaired swallow efficiency but was also associated with impaired swallow safety (aspiration). Based on these findings, even when aspiration is not observed during a FEES examination, it is presumed that severe pyriform sinus pooling could be predictive for or associated with aspiration that may or may not have been visualized. On these grounds, we suggest to pay specific attention to the presence of postswallow pharyngeal pooling during the swallowing assessment, in order to estimate the severity of OD and to design an appropriate OD management plan.

## Limitations of the Study

Our study has some limitations. Solid food boluses were not examined in the present study. In some HNC patients, the pyriform sinuses may be occluded from edema, fibrosis, and/or surgical changes. The cavity size of the pyriform sinuses could also be a determining factor in the risk analysis of aspiration, as well as the subjective visuoperceptual assessment of the amount of pooling. Moreover, the sample size was too small to allow detailed group stratification for all tumor locations, TNM classifications, and all single or combined oncologic treatment modalities. Therefore, it was not possible to analyze the potential effect of these parameters on the association between pooling and aspiration. However, the patient population included was a realistic representation of HNC patients consulting the multidisciplinary outpatient clinic for dysphagic complaints, which gives insight into the overall severity of swallowing impairment in this group. We hope to investigate these factors in a larger sample size in the future.

## Conclusion

Location (valleculae versus pyriform sinuses), liquid bolus consistency (thin versus thick liquid), and amount of postswallow pharyngeal pooling (no pooling, mild/moderate pooling, severe pooling) seem to have an influence on the probability of aspiration. Severe versus mild-to-moderate postswallow vallecular pooling of thick liquid was significantly associated to aspiration in HNC patients with OD, independent of the presence of pyriform sinus pooling, tumor stage, tumor location, or type of cancer treatment. Furthermore, this study showed a significant association between severe postswallow pyriform sinus pooling of thick liquid and aspiration. This association was independent of the presence of vallecular pooling, tumor stage, tumor location, or cancer treatment. Based on these findings, identification of location and amount of pooling during FEES evaluation should be carefully considered, even in the absence of aspiration during the examination.

## Electronic supplementary material

Below is the link to the electronic supplementary material.

Supplementary material 1 (DOCX 66 kb)

Supplementary material 2 (DOCX 66 kb)
