# Supplementary material for: Association Between Pharyngeal Pooling and Aspiration Using Fiberoptic Endoscopic Evaluation of Swallowing in Head and Neck Cancer Patients with Dysphagia
Source: Dysphagia. 2019 Mar 13;35(1):42–51. doi: 10.1007/s00455-019-09992-x (PMC6987057; doi:10.1007/s00455-019-09992-x)
Supplement: Supplementary file 1 — Supplementary material 1 (DOCX 66 kb) [file 455_2019_9992_MOESM1_ESM.docx]

Association between pharyngeal pooling and aspiration using fiberoptic endoscopic evaluation of swallowing in head and neck cancer patients with dysphagia

Dysphagia

Sorina R. Simon (MD)^1*^, Michelle Florie (MD)^1*^, Walmari Pilz (SLP, PhD)^1,2^, Bjorn Winkens (PhD)^3,4^, Naomi Winter^1^, Bernd Kremer (MD, PhD)^1,2^, Laura W.J. Baijens (MD, PhD)^1,2^

**these authors contributed equally to this work*

^1^Department of Otorhinolaryngology, Head and Neck Surgery, Maastricht University Medical Center, P.O. Box 5800, 6202 AZ Maastricht, The Netherlands;

^2^School for Oncology and Developmental Biology – GROW, Maastricht University Medical Center, Maastricht, The Netherlands;

^3^Department of Methodology and Statistics, Maastricht University, Maastricht, The Netherlands;

^4^Care and Public Health Research Institute – CAPHRI, Maastricht University Medical Center, Maastricht, The Netherlands.

E-mail address corresponding author: sr.simon@alumni.maastrichtuniversity.nl

**Table S1** Description of the fiberoptic endoscopic evaluation of swallowing outcome variables (as described in previous studies [20,21]

| **FEES ordinal outcome variable** | **Description** | **Scale^a,b^** |
| --- | --- | --- |
| Postswallow vallecular pooling | Pooling in the valleculae after the swallow | Three-point scale (range 0–2)  0 = no pooling  1 = mild to moderate pooling (filling of less than 50 % of the valleculae)  2 = severe pooling (filling of more than 50 % of the valleculae up to complete filling) |
| Postswallow pyriform sinus pooling | Pooling in the pyriform sinuses after the swallow | Three-point scale (range 0–2)  0 = no pooling  1 = mild to moderate pooling (filling of less than 50 % of the pyriform sinuses)  2 = severe pooling (filling of more than 50 % of the pyriform sinuses up to complete filling) |
| Aspiration | Bolus passing below the level of the vocal folds and entering the trachea | Two-point scale (range 0-1)  0 = no aspiration  1 = aspiration |

*FEES* fiberoptic endoscopic evaluation of swallowing

^a^ Lower scores refer to normal functioning; higher scores refer to more severe disability

^b^ A pooling score of 1 indicates mild to moderate pooling; a pooling score of 2 indicates severe pooling
