# Supplementary material for: Association Between Pharyngeal Pooling and Aspiration Using Fiberoptic Endoscopic Evaluation of Swallowing in Head and Neck Cancer Patients with Dysphagia
Source: Dysphagia. 2019 Mar 13;35(1):42–51. doi: 10.1007/s00455-019-09992-x (PMC6987057; doi:10.1007/s00455-019-09992-x)
Supplement: Supplementary file 2 — Supplementary material 2 (DOCX 66 kb) [file 455_2019_9992_MOESM2_ESM.docx]

Association between pharyngeal pooling and aspiration using fiberoptic endoscopic evaluation of swallowing in head and neck cancer patients with dysphagia

Dysphagia

Sorina R. Simon (MD)^1*^, Michelle Florie (MD)^1*^, Walmari Pilz (SLP, PhD)^1,2^, Bjorn Winkens (PhD)^3,4^, Naomi Winter^1^, Bernd Kremer (MD, PhD)^1,2^, Laura W.J. Baijens (MD, PhD)^1,2^

**these authors contributed equally to this work*

^1^Department of Otorhinolaryngology, Head and Neck Surgery, Maastricht University Medical Center, P.O. Box 5800, 6202 AZ Maastricht, The Netherlands;

^2^School for Oncology and Developmental Biology – GROW, Maastricht University Medical Center, Maastricht, The Netherlands;

^3^Department of Methodology and Statistics, Maastricht University, Maastricht, The Netherlands;

^4^Care and Public Health Research Institute – CAPHRI, Maastricht University Medical Center, Maastricht, The Netherlands.

E-mail address corresponding author: sr.simon@alumni.maastrichtuniversity.nl

**Table S2** Observer agreement levels (linearly weighted kappa) of the fiberoptic endoscopic evaluation of swallowing (FEES) outcome variables

| **FEES ordinal outcome variable** | **Interobserver agreement^a^** | **Intraobserver agreement^a^ (observer 1; observer 2)** |
| --- | --- | --- |
| Postswallow vallecular pooling | κ= 0.73 | κ= 0.76; 0.87 |
| Postswallow pyriform sinus pooling | κ= 0.71 | κ= 0.81; 0.84 |
| Aspiration | κ= 0.76 | κ= 0.81; 0.71 |

<0 less than chance agreement

0.01–0.20 slight agreement

0.21–0.40 fair agreement

0.41–0.60 moderate agreement

0.61–0.80 substantial agreement

0.81–0.99 almost perfect agreement

*FEES* fiberoptic endoscopic evaluation of swallowing

^a^ Kappa agreement (linearly weighted kappa coefficient of agreement)
